# Alarming Trends of Cesarean Section—Time to Rethink: Evidence From a Large-Scale Cross-sectional Sample Survey in India

**DOI:** 10.2196/41892

**Published:** 2023-02-13

**Authors:** Anuj Kumar Pandey, Mukesh Ravi Raushan, Diksha Gautam, Sutapa Bandyopadhyay Neogi

**Affiliations:** 1 Department of Health Research International Institute of Health Management Research New Delhi India

**Keywords:** cesarean section, surgical obstetric procedure, abdominal delivery, reproductive health service, women health services, obstetrics, health promotion

## Abstract

**Background:**

Cesarean section (CS) delivery rate has increased significantly both globally and in India, thereby posing a burden on overstretched health systems.

**Objective:**

This study plans to understand the factors associated with CS rate with an objective to (1) analyze the trends of CS delivery from 1998-99 to 2019-21 and (2) understand the proximate determinants of CS deliveries in India.

**Methods:**

Analysis of secondary data (National Family Health Survey) of a nationally representative sample of 230,870 women (year 2019-21) was undertaken to explore the trends, distribution, and determinants of CS deliveries in India and within states. Multivariable analyses were performed to determine the proximate variables associated with CS and elective CS. The relative interaction effect of confounding factors, such as number of antenatal care (ANC) visits, place of residence, and wealth status, on cesarean delivery was assessed. A composite index was generated using trust, support, and intimate partner violence variables (termed the partner human capital index [PHI]) to study its influence on CS deliveries, with a low PHI indicating abusive partner and a high PHI indicating supportive partner. Statewise spatial distribution of the most significantly associated factors, namely, wealth quintile and ANC checkups, were also analyzed.

**Results:**

The overall prevalence of CS was 21.50% (49,634/230,870) which had risen from 16.72% (2312/13,829) in 1998-99. The adjusted odds of CS deliveries were significantly higher among women who were highly educated (odds ratio [OR] 7.30, 95% CI 7.02-7.60; *P*<.001), had 4 or more ANC visits (OR 2.28, 95% CI 2.15-2.42; *P*<.001), belonging to the high-wealth quintile (OR 7.87, 95% CI 7.57-8.18; *P*<.001), and from urban regions. Increasing educational level of the head of the household (OR 3.05, 95% CI 2.94-3.16; *P*<.001) was also found to be a significant determinant of CS deliveries. The odds of selection of elective and emergency CS were also significantly higher among women from richer families (OR 1.66, 95% CI 1.25-2.21; *P*<.001) and those belonging to Christian religion (OR 1.67, 95% CI 1.14-2.43; *P*=.008). Adjusting the cesarean delivery by PHI, the odds of outcome were significantly higher among women with moderate and high PHI compared with those with low PHI (OR 1.46, 95% CI 1.36-1.56 and OR 1.61, 95% CI 1.49-1.74, respectively; *P*<.001 for both). The interaction effect result reiterates that women with more than 4 ANC checkups, high PHI, and belonging to the richer wealth quintile were more likely to undergo cesarean delivery (OR 22.22, 95% CI 14.99-32.93; *P*<.001) compared with those with no ANC visit, low PHI, and poorest women.

**Conclusions:**

The increasing trend of CS deliveries across India is raising concerns. Better education, wealth, and good support from the partner have been incriminated as the contributory factors. There is a need to institute proper monitoring mechanisms to assess the need for CS, especially when performed electively.

## Introduction

Cesarean section (CS) is a major life-saving surgical obstetric procedure that is highly effective in saving the lives of both the mother and the infant; however, it is recommended only for medically indicated causes [1,2]. For over a decade, there has been a rapid increase in CS delivery rates across the globe [3-5]. Globally, the number of cesarean births recorded each year is more than 18 million, accounting for approximately 19.1% of total births. These numbers have increased from just 7% in 1990 and are projected to increase to nearly one-third (29%) of all births by 2030 [1,6].

Both developed and developing countries fare similarly with respect to the prevalence of CS delivery (27.2% vs 20.9%). CS accounts for 7.3% of deliveries in Africa, whereas the rate increases to 40.5% in Latin America and the Caribbean [7]. The highest average annual rate of increase is observed in the regions of Asia (6.4%) [8]. In India, the proportion of cesarean deliveries has dramatically increased to 17% (2015-16) and 21.5% (2019-21) from just 3% in 1992-93 [9].

The rate of CSs performed has always been a subject of debate due to its variable need across the world that in turn is based on the nature of population, health care facility’s capacity to handle cases, availability of resources, and the clinical management protocols applied locally. Based on the available evidence, however, there is no justification for any region to have a CS rate higher than 10%-15% [10], regardless of their complexity or other characteristics. A systematic review concluded that at the population level, cesarean rates higher than 10% were not associated with reductions in maternal and newborn mortality rates [2]. Thus, CS must only be performed when medically indicated and in facilities equipped to treat surgical complications [11]. Rather than aiming for a certain rate, the World Health Organization (WHO) urges that every attempt should be made to provide CS only to women in need [10].

The *Lancet Commission on Surgery and Global Health* stated that surgical interventions are essential in bringing down the mortality and morbidity rates at all stages of life [12]. To achieve target 3.1 of the Sustainable Development Goals (SDG; ie, reducing the global maternal mortality ratio to <70 per 100,000 live births), collaborative efforts are required [13]. The aim of SDG-3 [14] is to ensure healthy lives and promote well-being, and therefore, it is essential to understand the geographical disparity and to explore the determinants of CS in India.

Besides clinical indications, the factors associated with increased rate of CS are demographic changes, social and educational advancements that have given rise to obstetrician’s preference for it, financial incentives, women’s request for cesarean delivery, women deferring pregnancy until they reach the end of their reproductive years, and inadequate training of physicians in vaginal delivery. Socioeconomic inequities appear to have created a pattern of underuse and overuse of CS based on income and levels of education [15-17].

According to the nationally representative survey, 1 in every 5 pregnant women in India had a CS even if they did not require it medically [8]. The rates in India have surpassed the WHO threshold of 15%, posing a serious public health risk. The rate of CS delivery is 47.4% in private facilities compared with 14.3% in public facilities [9]. One study reported that if private sector institutions in India had adopted the WHO’s 15% cesarean delivery rate standard, the number of preventable cesarean deliveries would have been 1.83 million, with potential cost savings of US $320.60 million [18].

Several studies have investigated factors contributing to CS [3,17,19-22]. Although the socioeconomic lopsidedness of CS deliveries in India toward urban and wealthier population is well established, some social factors (eg, partner’s human capital comprising factors such as any behavior within intimate relationships, trust, psychological abuse, and other controlling behavior) have not been explored thus far. Hence, this study is planned to understand the factors associated with CS rate with an objective to analyze the trends of CS delivery from 1998-99 to 2019-21 and to understand the proximate determinants of CS deliveries in India.

## Methods

### Data Source

**Figure 1:**
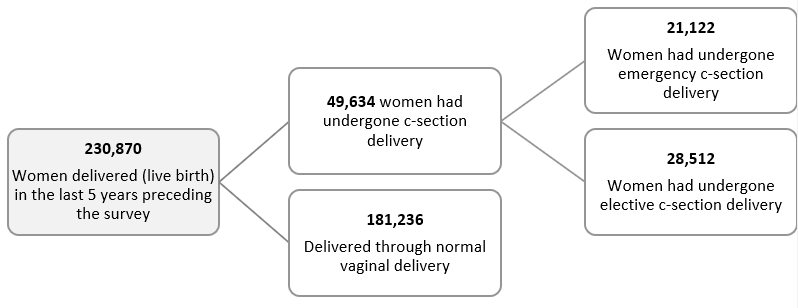
Survey participants descriptions of NHFS-5. C-section: cesarean section; NFHS: National Family Health Survey.

This study used data on cesarean delivery from a large-scale health survey: the National Family Health Survey (NFHS) [9]. The most recent round of the NFHS-5 was conducted from June 17, 2019, to April 30, 2021, covering 28 states and 8 union territories in 2 phases (International Institute of Population Studies [IIPS] and ICF 2022). The Demographic and Health Surveys (DHS) Program has collected, analyzed, and disseminated accurate and representative data on population, health, HIV, and nutrition through more than 400 surveys in over 90 countries, and the currently available version of NFHS-5 (7AFL) was used for the analysis. The other round of data was used to understand the level of CS delivery across states by years. As shown in Figure 1, a total of 230,870 women who had a live birth in last 5 years preceding the survey were included in the study. A total of 49,634 and 42,884 women had undergone CS in the NFHS-5 and NFHS-4, respectively.

### Variables

To ascertain the proximate variables associated with CS, the NFHS-5 results were considered. The primary outcome was CS in the last 5 years prior to the survey. Explanatory variables were those available from literature; these included individual- and community-level factors such as age, educational attainment of women, educational attainment of the head of the household, obstetric history, place of delivery, height of women, BMI of women, wealth index, place of residence, caste, religion, and region. Multimedia Appendix 1 provides an in-depth narrative and coded categories of the included variables.

A composite index was created using multiple dichotomous variables (Multimedia Appendix 1), named as the partner human capital index (PHI; Cronbach *α*=.80), to study the partner’s influence on CS deliveries. The generated scores were categorized using percentile value into “low,” indicating abusive, suspicious, and inhuman partner; “moderate” and “high,” which implies highly supportive and caring partner. A total of 24,216 women provided information about the included variables for PHI, and thus a separate stepwise reverse regression analysis was performed by keeping the sample as 24,216 to identify the determinants.

### Missing Values

Of the 232,920 observations of women who had an institutional delivery during the 5 years preceding the survey, in 24% (55,901/232,920) of cases, the number of antenatal care (ANC) visits was missing. We assumed that the number of ANC visits is an important parameter that is necessary to detect high-risk pregnancy and also influence the decision for CS. Hence, we used the single imputation technique. The missing information on ANC visits was imputed based on baseline nonmissing background characteristics of women, namely, caste and region.

### Statistical Analysis

Analyses were performed to observe the trend in the frequency and distribution of CS over the past 15 years. This was conducted with regard to place of residence, type of health facilities, and need of CS. CS data for the periods 2005-06, 2015-16, and 2019-21 were graphically plotted to assess statewise distribution. We also studied the statewise spatial distribution of 2 most significantly associated factors, namely, “wealth quintile” (which was categorized as poor, middle, and richest using percentile values) and “ANC checkups” (which was categorized as no ANC, 1 ANC, or >4 ANCs using percentile), that have emerged from the multivariable analyses

Analyses were performed to explore the association of primary outcome and the explanatory variables. The initial bivariate analysis was conducted with *χ*^2^ test for ordered categorical variables. The list of confounding factors for unadjusted and adjusted regression models was screened based on bivariate analysis if the differences among categories were higher than 5%. Those with a significant difference (*P*<.05) and those biologically plausible were selected for the adjusted analysis.

Multivariable analyses were performed to determine the proximate variables associated with CS and later to ascertain the proximate variables associated with elective CS. The regression results are presented as odds ratio (OR) at 95% CI. The stepwise reverse regression models were used to maintain the same sample size for variables with the smaller number of cases, such as information available at partner’s level. The list of variables for assessing the interaction was chosen based on the differences among categories (*P*<.05).

### Ethics Approval

All data are available in the public domain that could be accessed followed registration on the website and hence there are no ethical implications [23].

## Results

### Prevalence of Cesarean Delivery and Associated Attributes

**Figure 2:**
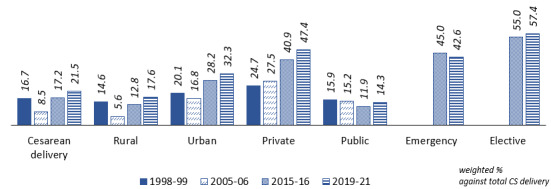
Percentage of women delivered through caesarean section in India (1998-2021). CS: cesarean section.

A total of 230,870 women who delivered in the period spanning 5 years from the date of survey were included in this study, which included 707 districts and 36 states/union territories of India. Of these, 21.50% (49,634/230,870) delivered through CS, which had risen from 16.72% (2312/13,829) in 1998-99 (Figure 2).

The prevalence of CS deliveries was more in urban areas (32.3% vs rural 17.6%) and in private facilities (47.0% vs 14.3% in public facilities; Table 1). Furthermore, it is evident from Figure 2 that CS deliveries have increased manifolds at private facilities as against the stagnant rates in public health care facilities. Of the total deliveries reported in India during 2019-21, 12.35% (28,512/230,870) were elective CS, whereas 9.15% (21,122/230,870) were emergency CS (Figure 2).

**Figure 3:**
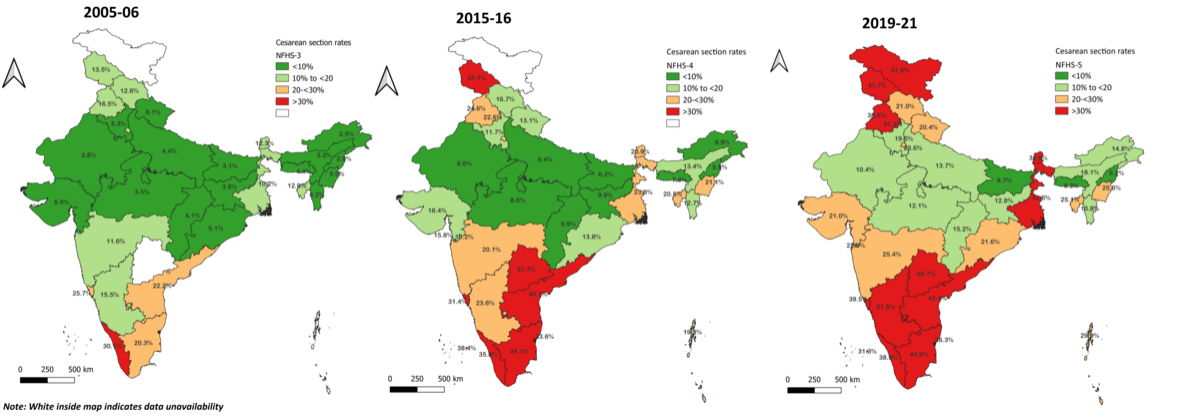
Spatial distribution of percentage of caesarean section delivery across Indian States. NFHS: National Family Health Survey.

A statewise comparison (Figure 3) shows an overall change in the CS deliveries over the years across all states/union territories with a slight skewness toward the southern and extreme northern states. It was also evident that 14.3% (101/707) of the districts in 2019-21 had CS rate more than the WHO-recommended cutoff of <15% (101/641 districts, 15.8%) and that in 2015-16.

The prevalence of CS was higher among those who were older than 25 years (70.1%), those who were more educated (40.67%), with low family size (27.29%), from urban areas (32.26%), and general caste (28.44%). The probability of CS among women who are residing in the northern and southern parts of India was higher (70.85%; Table 1), which is also evident from the spatial distribution of CS (Figure 3). The probability was more among women with 1 parity (31.96%), those having 4 or more ANC visits (27.32%), those with tall stature (23.92%), who were overweight or obese (81.83%), those with mild (21.42%) or no anemia (23.39%), and those belonging to the higher wealth quintile (ie, richest; 39.12%; Table 1).

**Table 1:** Socioeconomic and demographic factors associated with type of delivery among women in India 5 years prior to the 2019-21 survey (n=232,920).

Background characteristics		Normal (n=181,236), n/N (%)	Cesarean section (n=49,634), n/N (%)	Total deliveries unweighted (n=232,920), n	Cesarean delivery			
					Unadjusted odds ratio (95% CI)	Adjusted odds ratio (95% CI)^a^		
**Age of women (years)**								
	15-19	5032/6115 (82.29)	1083/6115 (17.71)	5461	1^b^	1		
	20-24	56,564/69,948 (80.87)	13,384/69,948 (19.13)	66,485	1.10^c^ (1.03-1.18)	1 (1.00-1.00)		
	25-29	72,880/92,834 (78.51)	19,954/92,834 (21.49)	92,448	1.27^d^ (1.19-1.36)	1.04 (0.97-1.12)		
	30-34	32,181/42,854 (75.09)	10,673/42,854 (24.91)	45,587	1.54^d^ (1.44-1.65)	1.18^d^ (1.10-1.26)		
	>35	14,580/19,119 (76.26)	4539/19,119 (23.74)	22,939	1.45^d^ (1.34-1.56)	1.43^d^ (1.33-1.53)		
**Educational level of women**								
	Illiterate	45,484/49,306 (92.3)	3822/49,306 (7.8)	51,210	1	1		
	Primary	24,847/28,434 (87.4)	3587/28,434 (12.6)	30,081	1.72^d^ (1.64-1.80)	1.71^d^ (1.63-1.80)		
	Secondary	89,487/1,17,031 (76.5)	27,544/1,17,031 (23.5)	1,19,864	3.66^d^ (3.53-3.80)	3.51^d^ (3.39-3.64)		
	Secondary and higher	21,419/36,099 (59.3)	14,680/36,099 (40.7)	31,765	8.16^d^ (7.84-8.48)	7.30^d^ (7.02-7.60)		
**Educational level of head of household**								
	Illiterate	61,999/72,847 (85.11)	10,847/72,847 (14.89)	73,831	1	1		
	Primary	34,594/43,018 (80.42)	8424/43,018 (19.58)	43,293	1.39^d^ (1.35-1.44)	1.37^d^ (1.33-1.41)		
	Secondary	72,442/95,281 (76.03)	22,839/95,281 (23.97)	97,394	1.80^d^ (1.76-1.85)	1.69^d^ (1.64-1.73)		
	Secondary and higher	12,201/19,725 (61.85)	7524/19,725 (38.15)	18,402	3.53^d^ (3.40-3.65)	3.05^d^ (2.94-3.16)		
**Household size**								
	1-4	42,928/59,040 (72.7)	16,112/59,040 (27.3)	60,455	1	—^e^		
	5-10	124,380/155,114 (80.2)	30,734/155,114 (19.8)	1,57,153	0.66^d^ (0.64-0.67)	—		
	>11	13,928/16,716 (83.3)	2787/16,716 (16.7)	15,312	0.53^d^ (0.51-0.56)	—		
**Place of residence**								
	Urban	41,682/61,528 (67.74)	19,846/61,528 (32.26)	47,199	1	1		
	Rural	139,554/169,342 (82.41)	29,788/169,342 (17.59)	1,85,721	0.45^d^ (0.44-0.46)	0.48^d^ (0.47-0.49)		
**Religion**								
	Hindu	144,024/183,338 (78.6)	39,315/183,338 (21.4)	1,71,055	1	—		
	Muslim	30,132/37,495 (80.4)	7363/37,495 (19.6)	33,522	0.90^d^ (0.87-0.92)	—		
	Christian	3435/4784 (71.8)	1348/4784 (28.2)	18,851	1.44^d^ (1.35-1.53)	—		
	Others	3645/5253 (69.4)	1608/5253 (30.6)	9492	1.62^d^ (1.52-1.72)	—		
**Caste^f^**								
	Scheduled caste	47,620/58,923 (80.82)	11,303/58,923 (19.18)	52,729	1	—		
	Scheduled tribe	22,563/25,821 (87.38)	3258/25,821 (12.62)	49,569	0.61^d^ (0.58-0.63)	—		
	Other backward classes	79,628/102,213 (77.9)	22,585/102,213 (22.1)	91,122	1.20^d^ (1.17-1.23)	—		
	None	31,425/43,913 (71.56)	12,488/43,913 (28.44)	39,500	1.67^d^ (1.63-1.72)	—		
**Region**								
	Southern	22,030/38,139 (57.76)	16,109/38,139 (42.24)	29,269	1	—		
	North	10,137/14,199 (71.39)	4062/14,199 (28.61)	64,344	0.55^d^ (0.53-0.57)	—		
	West	35,175/43,620 (80.64)	8445/43,620 (19.36)	24,663	0.33^d^ (0.32-0.34)	—		
	Northeastern	6915/8388 (82.44)	1473/8388 (17.56)	45,227	0.29^d^ (0.27-0.31)	—		
	Eastern	49,810/60,323 (82.57)	10,513/60,323 (17.43)	34,222	0.29^d^ (0.28-0.30)	—		
	Central	57,169/66,201 (86.36)	9032/66,201 (13.64)	35,195	0.22^d^ (0.21-0.22)	—		
**Wealth index**								
	Poorest	52,603/56,771 (92.7)	4167/56,771 (7.3)	63,406	1	1		
	Poorer	42,632/50,170 (85)	7538/50,170 (15)	54,463	2.23^d^ (2.14-2.32)	2.23^d^ (2.14-2.32)		
	Middle	34,304/45,101 (76.1)	10,798/45,101 (23.9)	45,083	3.97^d^ (3.82-4.13)	3.86^d^ (3.71-4.01)		
	Richer	29,582/42,505 (69.6)	12,923/42,505 (30.4)	39,094	5.51^d^ (5.31-5.73)	5.33^d^ (5.13-5.54)		
	Richest	22,114/36,323 (60.9)	14,208/36,323 (39.1)	30,874	8.11^d^ (7.81-8.42)	7.87^d^ (7.57-8.18)		
**Partner human capital index^g^**								
	Low	8968/10,773 (83.25)	1805/10,773 (16.75)	10,450	1	1		
	Moderate	7903/10,360 (76.29)	2457/10,360 (23.71)	10,959	1.54^d^ (1.44-1.65)	1.52^d^ (1.42-1.63)		
	High	4826/6496 (74.29)	1670/6496 (25.71)	6896	1.72^d^ (1.59-1.85)	1.68^d^ (1.55-1.81)		
**Parity**								
	1	40,799/59,962 (68.04)	19,163/59,962 (31.96)	59,620	1	1		
	2	67,786/90,655 (74.77)	22,869/90,655 (25.23)	88,770	0.72^d^ (0.70-0.73)	0.70^d^ (0.68-0.72)		
	3+	72,651/80,253 (90.53)	7602/80,253 (9.47)	84,530	0.22^d^ (0.22-0.23)	0.23^d^ (0.22-0.24)		
**Number of antenatal care visits**								
	No visit	9303/10,712 (86.84)	1409/10,712 (13.16)	11,462	1	1		
	1-4 visits	86,907/103,171 (84.24)	16,264/103,171 (15.76)	1,07,789	1.24^d^ (1.17-1.31)	1.19^d^ (1.12-1.26)		
	>4 visits	85,027/116,987 (72.68)	31,960/116,987 (27.32)	1,13,669	2.48^d^ (2.34-2.63)	2.28^d^ (2.15-2.42)		
**Height of women (cm)^h^**								
	150.0-155.0 (average)	60,184/76,136 (79.05)	15,952/76,136 (20.95)	77,673	1	1		
	<150.0 (short)	68,477/85,357 (80.22)	16,880/85,357 (19.78)	85,613	0.93^d^ (0.91-0.95)	—		
	155.1-239.9 (tall)	47,071/61,867 (76.08)	14,796/61,867 (23.92)	63,856	1.19^d^ (1.16-1.22)	—		
	Refused/not present	5504/7509 (73.29)	2006/7509 (26.71)	5778	—	—		
**BMI of women (kg/m^2^)**								
	<18.40 (underweight)	35,688/41,276 (86.46)	5587/41,276 (13.54)	1,43,636	1	1		
	18.41-24.99 (normal)	114,852/140,946 (81.49)	26,094/140,946 (18.51)	42,431	0.68^d^ (0.66-0.70)	0.70^d^ (0.68-0.73)		
	24.5-29.9 (overweight)	25,501/38,716 (65.87)	13,215/38,716 (34.13)	38,070	2.27^d^ (2.21-2.32)	2.13^d^ (2.08-2.18)		
	>30.0 (obesity)	5195/9933 (52.3)	4738/9933 (47.7)	8783	3.99^d^ (3.83-4.16)	3.58^d^ (3.43-3.73)		
**Anemia level (g/dl)^h^**								
	5.0-6.9 (severe)	4312/5092 (84.67)	780/5092 (15.33)	5512	1	1		
	7-9.9 g/dl (moderate)	55,503/68,565 (80.95)	13,062/68,565 (19.05)	69,118	1.30^d^ (1.20-1.41)	1.33^d^ (1.23-1.44)		
	10-10.9 g/dl (mild)	45,917/58,432 (78.58)	12,515/58,432 (21.42)	58,350	1.51^d^ (1.39-1.63)	1.51^d^ (1.39-1.63)		
	>10.9 g/dl (no anemic)	67,581/88,208 (76.61)	20,628/88,208 (23.39)	91,403	1.69^d^ (1.56-1.82)	1.62^d^ (1.49-1.75)		
	Refused/not present	7923/10,573 (74.94)	2650/10,573 (25.06)	8537	—	—		
**Place of delivery^i^**								
	Public	122,484/142,943 (85.7)	20,459/142,943 (14.3)	1,50,299	1	1		
	Private	32,904/62,079 (53)	29,175/62,079 (47)	51,012	5.31^d^ (5.19-5.42)	5.26^d^ (5.14-5.38)		

^a^Controlled for size of the household, religion, caste, and region.

^b^Reference category.

^c^*P*<.01.

^d^*P*<.001.

^e^Variables not considered for model analysis.

^f^The constitutional classification of caste was recoded as follows: scheduled caste, scheduled tribe, other backward caste, and none (ie, none of scheduled caste, scheduled tribe, and other backward class).

^g^The n for partner capital index is 21,697 and 5932 for normal delivery and cesarean section, respectively (N=27,629). For unweighted, n=28,305.

^h^A total of 7509 and 10,573 women refused to participate in height and hemoglobin measurement, respectively. These women were not included in regressions.

^i^A total of 31,609 women delivered in home.

### Logit Regression Analysis

Logit regression adjusted for caste, religion, and region was performed, which found that the odds of CS were significantly higher among women having secondary education (OR 3.51, 95% CI 3.39-3.64) or higher (OR 7.30; 95% CI 7.02-7.60) compared with those who were illiterate (Table 1). Increasing educational attainment of the head of the household was also found to increase the odds of having CS (OR 3.05, 95% CI 2.94-3.16). The odds of cesarean delivery among women with 4 or more ANC visits (OR 2.28, 95% CI 2.15-2.42), those belonging to the high-wealth quintile (OR 7.87, 95% CI 7.57-8.18), and from urban region were higher compared with their counterparts with less than 4 ANC visits, who were poor, and residing in rural region, respectively (*P*<.001; Table 1). Table 1 presents information on other predictors of CS deliveries.

The dynamics of confounding factors was clearer after including the partner’s characteristics to cesarean delivery, indicating closer association with the outcome. The null model of reverse regression indicated that the odds of cesarean delivery was 0.32 (95% CI 0.31-0.33; *P*<.001). The odds of outcome were significantly higher among women with moderate (OR 1.46, 95% CI 1.36-1.56) and high (OR 1.61, 95% CI 1.49-1.74) PHIs. After adjusting for other background characteristics of women, the strength of the association reduced (OR 1.24, 95% CI 1.14-1.35) but remained statistically significant (Table 2).

The available information on type of CS in terms of elective and emergency surgery was analyzed as presented in Multimedia Appendix 2. The odds of elective cesarean delivery were higher among women belonging to the higher wealth status (OR 1.66, 95% CI 1.25-2.21), women belonging to Christian religion (OR 1.67, 95% CI 1.14-2.43), and those with lower parity (Multimedia Appendix 2).

The relative interaction effects of confounding factors such as number of ANC visits, place of residence, wealth status, and PHI on cesarean delivery were estimated and shown in Tables 3 and 4. The odds of CS among women with 4 or more ANC visits increased from 2.92 (95% CI 2.63-3.25) to 14.86 (95% CI 13.49-16.37) in those belonging to the poorest and richest wealth quintiles, respectively (*P*<.001).

The odds of CS were 3.14 (95% CI 2.56-3.85) among women with 4 or more ANC visits and higher PHI compared with their reference category (ie, no ANC visits and low PHI, respectively). The combined effect of place of residence and educational attainment remained statistically significant and indicated that urban educated women were more likely to have CS than rural educated women. Among women with 4 or more ANC visits, the odds of cesarean delivery starkly increased among women with 12 or more years of education, especially in urban regions (OR 10.99, 95% CI 8.71-13.87) compared with urban illiterate women in India (OR 2.83, 95% CI 2.18-3.67; *P*<.001). Women with high PHI, belonging to the richest wealth quintile, and having 4 or more ANC visits were more likely to undergo cesarean delivery (OR 22.22, 95% CI 14.99-32.93) compared with those with low PHI, no visits, and belonging to the poorest wealth status (Tables 3 and 4).

**Figure 4:**
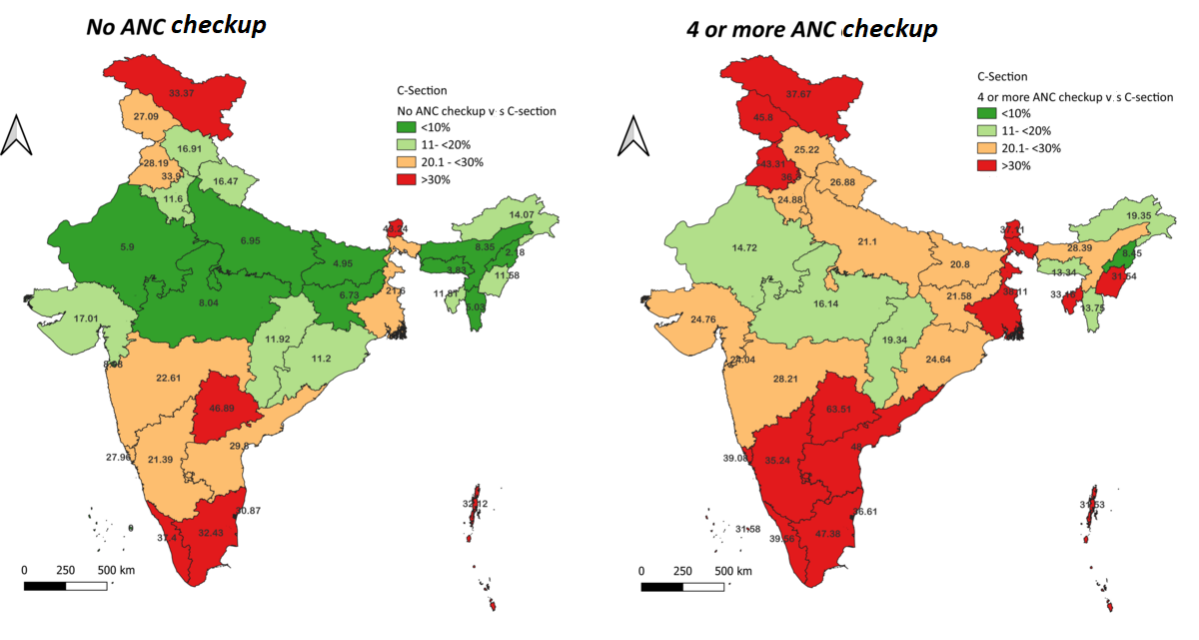
Spatial distribution of percentage of c-section (cesarean section) delivery among women with no ANC check-ups versus 4 or more ANC check-ups across Indian States 2019-21. ANC: antenatal checkup; C-section: cesarean section.

**Figure 5:**
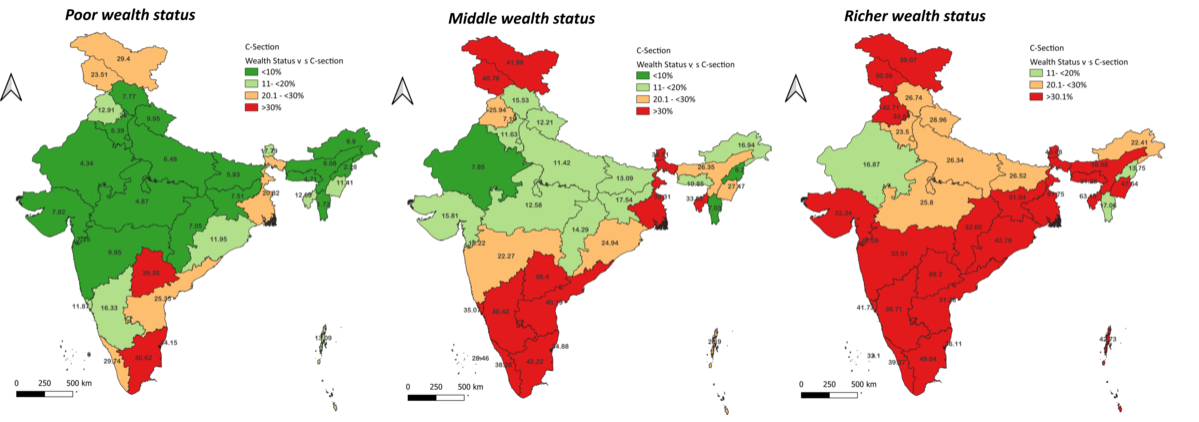
Spatial distribution of percentage of cesarean section (c-section) delivery among women belonging to different wealth status across Indian States 2019-21. C-section: cesarean section.

Categorizing statewise distribution of CS deliveries by ANC visits reiterated the regional disparities. It was found that women from southern and northern states have higher CS deliveries (higher than the WHO’s cutoff) irrespective of the ANC status (Figure 4). Likewise, regardless of the region, women belonging to the wealthier households underwent CS deliveries (Figure 5).

**Table 2:** Result of regression models for cesarean delivery for partner human capital index (n=24,216).

Background characteristics			Null model (ie, model without exposure variable)		Odds for cesarean section adjusted for partner human capital index (95% CI)		Odds of cesarean section adjusted for partner human capital index and other (95% CI)^a^
**Husband human capital index**							
	Low	—^b^		1^c^		1	
	Moderate	—		1.46^d^ (1.36-1.56)		1.15^d^ (1.07-1.24)	
	High	—		1.61^d^ (1.49-1.74)		1.24^d^ (1.14-1.35)	
**Educational attainment of women**							
	Illiterate	—		—		1	
	Primary	—		—		1.30^d^ (1.12-1.51)	
	Secondary	—		—		1.57^d^ (1.40-1.77)	
	Secondary and higher	—		—		1.49^d^ (1.29-1.71)	
**Number of antenatal care visit**							
	No visit	—		—		1	
	1-4 visits	—		—		0.87 (0.72-1.07)	
	>4 visits	—		—		1.12 (0.92-1.36)	
**Parity**							
	1	—		—		1	
	2	—		—		0.75^d^ (0.70-0.81)	
	3+	—		—		0.43^d^ (0.39-0.48)	
**Place of delivery**							
	Public	—		—		1	
	Private	—		—		3.94^d^ (3.67-4.22)	
**Wealth index**							
	Poorest	—		—		1	
	Poorer	—		—		1.70^d^ (1.50-1.93)	
	Middle	—		—		2.54^d^ (2.24-2.88)	
	Richer	—		—		2.58^d^ (2.27-2.93)	
	Richest	—		—		2.47^d^ (2.15-2.83)	
Null model			0.32^d^ (0.31-0.33)		—		—

^a^Religion, caste, region, and household size were controlled.

^b^Variables not considered for model analysis.

^c^Reference category.

^d^*P*<.001.

**Table 3:** Result of logit regression models for cesarean delivery with interaction between covariates (n=28,305)^a^.

Covariates				Number of antenatal care visit							Place of residence: urban							Place of residence: rural							
				No visits, odds ratio (95% CI)		1-4 visits, odds ratio (95% CI)		4 or more visits, odds ratio (95% CI)		No visits, odds ratio (95% CI)			1-4 visits, odds ratio (95% CI)		4 or more visits, odds ratio (95% CI)		No visits, odds ratio (95% CI)			1-4 visits, odds ratio (95% CI)		4 or more visits, odds ratio (95% CI)			
**Place of residence**																									
		Urban	1 (1.00-1.00)		1.16^b^ (1.08-1.25)		1.96^b^ (1.83-2.11)		—^c^			—		—		—			—		—				
		Rural	0.42^b^ (0.39-0.46)		0.53^b^ (0.49-0.56)		1.22^b^ (1.13-1.30)		—			—		—		—			—		—				
**Wealth index**																									
		Poorest	1 (1.00-1.00)		1.33^b^ (1.20-1.47)		2.92^b^ (2.63-3.25)		1 (1.00-1.00)			0.7 (0.49-1.01)		1.97^b^ (1.39-2.79)		0.50^b^ (0.37-0.69)			0.70^d^ (0.52-0.95)		1.52^e^ (1.13-2.05)				
		Poorer	2.46^b^ (2.18-2.78)		2.80^b^ (2.53-3.09)		5.50^b^ (4.98-6.07)		1.73^e^ (1.21-2.48)			1.62^e^ (1.18-2.22)		3.12^b^ (2.29-4.26)		1.25 (0.92-1.70)			1.47^d^ (1.09-1.98)		2.88^b^ (2.14-3.87)				
		Middle	3.92^b^ (3.47-4.43)		4.81^b^ (4.36-5.30)		8.97^b^ (8.14-9.88)		1.94^b^ (1.39-2.71)			2.72^b^ (2.01-3.68)		4.85^b^ (3.59-6.54)		2.13^b^ (1.56-2.90)			2.51^b^ (1.86-3.37)		4.72^b^ (3.51-6.34)				
		Richer	5.62^b^ (4.96-6.37)		6.51^b^ (5.90-7.19)		11.31^b^ (10.27-12.46)		2.71^b^ (1.96-3.74)			4.06^b^ (3.01-5.48)		6.16^b^ (4.58-8.29)		3.19^b^ (2.33-4.37)			3.13^b^ (2.32-4.21)		5.85^b^ (4.35-7.86)				
		Richest	10.71^b^ (9.41-12.19)		10.08^b^ (9.12-11.14)		14.86^b^ (13.49-16.37)		6.68^b^ (4.88-9.15)			5.92^b^ (4.39-7.97)		8.27^b^ (6.15-11.12)		4.11^b^ (2.93-5.75)			4.55^b^ (3.37-6.14)		6.95^b^ (5.15-9.37)				
**Partner’s human capital**																									
		Low	1 (1.00-1.00)		1.28^d^ (1.04-1.57)		3.14^b^ (2.56-3.85)		1 (1.00-1.00)			1.06 (0.60-1.89)		1.80^d^ (1.02-3.17)		0.43^e^ (0.23-0.81)			0.58 (0.33-1.02)		1.08 (0.62-1.89)				
		Moderate	1.80^b^ (1.40-2.32)		1.74^b^ (1.42-2.13)		4.33^b^ (3.55-5.29)		1.88 (0.93-3.83)			1.67 (0.94-2.96)		2.89^b^ (1.64-5.06)		0.45^d^ (0.23-0.90)			0.7 (0.40-1.23)		1.5 (0.86-2.62)				
		High	1.71^b^ (1.29-2.28)		1.90^b^ (1.54-2.35)		4.81^b^ (3.93-5.90)		2.03 (0.91-4.53)			2.31^e^ (1.30-4.13)		3.21^b^ (1.83-5.65)		0.84 (0.43-1.66)			0.68 (0.39-1.21)		1.54 (0.88-2.70)				
**Women’s education**																									
	Illiterate		—		—		—		1 (1.00-1.00)			1.45^e^ (1.13-1.86)		2.83^b^ (2.18-3.67)		0.53^b^ (0.41-0.68)			0.79^d^ (0.62-1.00)		1.77^b^ (1.39-2.25)				
	Primary		—		—		—		1.97^b^ (1.45-2.68)			2.27^b^ (1.76-2.92)		3.50^b^ (2.73-4.49)		0.88 (0.67-1.15)			1.23 (0.97-1.56)		2.54^b^ (2.00-3.24)				
	Secondary		—		—		—		3.57^b^ (2.79-4.57)			4.33^b^ (3.43-5.48)		5.93^b^ (4.70-7.48)		2.07^b^ (1.63-2.63)			2.46^b^ (1.95-3.10)		4.24^b^ (3.36-5.34)				
	Secondary education and higher		—		—		—		9.14^b^ (6.99-11.96)			8.61^b^ (6.79-10.91)		10.99^b^ (8.71-13.87)		4.66^b^ (3.59-6.04)			4.67^b^ (3.69-5.91)		7.69^b^ (6.09-9.71)				

^a^Religion, caste, and region were controlled.

^b^*P*<.001.

^c^Variables not considered for model analysis.

^d^*P*<.05.

^e^*P*<.01.

**Table 4:** Result of logit regression models for cesarean delivery with interaction between partner’s human capital, wealth index, and access to available health services in terms of number of antenatal care visits in India (n=28,305)^a^.

Wealth index	Partner human capital: low, odds ratio (95% CI)				Partner human capital: moderate, odds ratio (95% CI)				Partner human capital: high, odds ratio (95% CI)		
	No visits, odds ratio (95% CI)	1-4 visits, odds ratio (95% CI)	4 or more visits, odds ratio (95% CI)	No visits, odds ratio (95% CI)		1-4 visits, odds ratio (95% CI)	4 or more visits, odds ratio (95% CI)	No visits, odds ratio (95% CI)		1-4 visits, odds ratio (95% CI)	4 or more visits, odds ratio (95% CI)
Poorest	1 (1.00-1.00)	0.98 (0.64-1.49)	3.06^b^ (1.99-4.71)	0.96 (0.50-1.83)		1.06 (0.68-1.65)	2.88^b^ (1.85-4.47)	0.15^c^ (0.03-0.68)		1.14 (0.71-1.84)	2.28^d^ (1.38-3.76)
Poorer	1.93^c^ (1.14-3.28)	2.82^b^ (1.89-4.19)	5.46^b^ (3.63-8.20)	2.34^d^ (1.37-4.01)		2.67^b^ (1.78-4.01)	5.30^b^ (3.53-7.96)	2.43^d^ (1.29-4.60)		3.88^b^ (2.55-5.89)	6.55^b^ (4.28-10.03)
Middle	3.86^b^ (2.24-6.63)	4.61^b^ (3.10-6.87)	8.57^b^ (5.74-12.80)	6.64^b^ (4.04-10.89)		5.48^b^ (3.67-8.17)	12.02^b^ (8.15-17.72)	6.35^b^ (3.62-11.14)		4.55^b^ (2.95-7.02)	10.67^b^ (7.15-15.91)
Richer	4.87^b^ (2.71-8.78)	6.57^b^ (4.37-9.86)	9.68^b^ (6.51-14.40)	6.87^b^ (4.08-11.56)		7.79^b^ (5.22-11.62)	13.42^b^ (9.11-19.76)	10.19^b^ (5.49-18.91)		8.34^b^ (5.47-12.70)	12.98^b^ (8.74-19.27)
Richest	6.40^b^ (3.08-13.33)	6.54^b^ (4.24-10.09)	12.03^b^ (8.06-17.95)	8.41^b^ (4.90-14.43)		10.59^b^ (7.03-15.95)	15.31^b^ (10.41-22.51)	14.02^b^ (7.64-25.74)		12.31^b^ (8.02-18.89)	22.22^b^ (14.99-32.93)

^a^Religion, caste, and region were controlled.

^b^*P*<.001.

^c^*P*<.05.

^d^*P*<.01.

## Discussion

### Principal Findings

Analysis of secondary data of a nationally representative sample of 230,870 women nested within 707 districts from 36 states/union territories of India demonstrated the increasing prevalence of CS deliveries in India during the past decades, that is, from 8.5% (4777/56,438) in 2005-06 to 21.5% (49,634/2,30,870) in 2019-21. The likelihood of CS was more among educated families with richer wealth status, those residing in urban areas, and those having better contact with health systems, as evident from the increased number of ANC visits. Good family support expressed by partner’s human capital resulted in greater odds of CS with no interaction with wealth status, education, or better availability of services in urban areas.

These findings are in line with a study that reported an upward trajectory of CS deliveries globally and in India [3,24]. State-specific prevalence in India vary from a low of 5.2% (in Nagaland) to a high of 60.7% (Telangana). In particular, our study reported increasing prevalence of CS deliveries across all the states/union territories among the wealthier population, which is similar to figures reported previously [24]. Furthermore, southern states with better health indicators have a greater preponderance of CS [6].

A significant increase in CS deliveries is being reported from private facilities (from 20% in 1998-99 to 47% in 2019-20). The literature reports similar findings [25-28], which could be associated with willingness to pay, especially among women belonging to families with wealthier status. An increase in CS rates could be attributed to physician’s choice in private sector [15], but it has been established that the probability of CS delivery and elective CS increases with better wealth quintiles [28-33]. Our study also echoes similar findings. This is in contrast to a global study across 57 developed and developing countries that reported that there is a poor correlation between income inequality and absolute wealth-related inequality in CS deliveries [34].

Further, affluent women have a greater likelihood of CS by choice, probably because of perceived lower risks [35]. This corroborates with our findings wherein wealth status independently influences the odds of having CS deliveries irrespective of the place of residence, ANC check-up status, or PHI. Similarly, better access to health services promotes CS. This is evident from an increase in the likelihood of CS births with the increase in the number of ANC visits or early initiation of ANC checkups [36,37], similar to our study.

However, there are conflicting results from studies that looked at the relationship between maternal education level and CS delivery. Although most of the studies from Bangladesh [25], Thailand [38], Pakistan [39], and India [24,40] reported a strong association of formal education with CS deliveries, the one from Egypt found no significant association [41]. Improved autonomy and capability to take decisions probably explain increased CS among educated women [41,42]. Along similar lines, education levels among heads of household also influence decisions for CS. Concerns around medical malpractices or viewing CS as a measure to prevent any mishappening could be the possible reasons that motivate families to support CS [43-45].

Access to equitable health services has generated a lot of discussions around social factors. Available studies have explored the relationship between relation with partner and violence and negative health outcomes [44-46]; however, we could not find any literature on its association with CS. To explore it further, we developed an index that comprehensively captures the attitude and behavioral issues toward the partners. This measure assumes importance when we attempt to understand the reproductive health issues beyond the medical lens.

### Strengths

Data from a large nationally representative sample collected using a scientific methodology is a strength of this analysis. Although several reports are already available on CS, this study provides the most recent data of a country as large as India. The findings underscore the growing concerns around CS. Besides, it highlights an important parameter of partner human capital, a composite index encompassing attitudinal and behavioral factors, that is rarely discussed in the context of CS. Imputation of a key exposure variable, 4 ANC visits, enabled us to overcome the gap resulting from missing data, thus paving the way for a more robust analysis.

### Limitations

Despite the fact that our study has numerous methodological and conceptual strengths, it also has certain limitations. The study captures information for a reference period of 5 years preceding the survey. Although self-reports on CS might not have been affected, other variables such as number of ANC visits pertaining to CS births and reporting of partner human capital may have suffered from a long recall period. We also sought to adjust for individual risk factors for CS, even after imputing for the missing values and excluding variables with more than 25% (25/100) missing values, but there are possibilities that we may have missed some essential predictors or determinants.

### Conclusions

The study reiterates the increasing trend of CS deliveries across India, thereby raising concerns. Better education, wealth, and social factors have been incriminated as the contributory factors. There is a need to institute proper monitoring mechanisms to assess the need for CS, especially when performed electively. Improved awareness about the obstetric dangers and postpartum complications of cesarean deliveries over normal deliveries along with strategical implementation of government initiatives can help us take a rational decision on CS deliveries. As we prioritize increasing access to services for better health gains, this turns out to be an example where better access predisposes to overmedicalization. Whereas low CS in underdeveloped communities can be a concern, the potential for medically unnecessary overuse of CS delivery as income, education, and PHI raises is a different set of issues that demands for targeted health policy interventions to achieve more appropriate use of CS among richer and more educated women.

## Appendix

Multimedia Appendix 1 Description and coding categories of variables used in analysis.

Multimedia Appendix 2 Socio-economic and demographic factors associated with type of delivery (elective versus emergency caesarean delivery) among women 5 years prior to survey, 2019-21, India.

## Abbreviations

ANC: antenatal checkup
CS: cesarean section
DHS: Demographic Health Survey
IIPS: International Institute of Population Studies
NFHS: National Family Health Survey
OR: odds ratio
PHI: partner human capital index
SDG: Sustainable Development Goals
WHO: World Health Organization

## Data Sharing

The supporting data of this study are available in the Demographic and Health Survey program [23].
