# Supplementary material for: Alarming Trends of Cesarean Section—Time to Rethink: Evidence From a Large-Scale Cross-sectional Sample Survey in India
Source: J Med Internet Res. 2023 Feb 13;25:e41892. doi: 10.2196/41892 (PMC9972201; doi:10.2196/41892)
Supplement: Multimedia Appendix 1 [file jmir_v25i1e41892_app1.docx]

**Multimedia Appendix 1.** Description and coding categories of variables used in analysis.

| Variable | Description and coding categories |
| --- | --- |
| Age | The age of women was recoded into 4 categories: 15-19, 20-29, 30-34, and <35 years |
| Partner human capital index | The information available in the data were converted into a binary value. For instance, if the respondent reported “husband/partner jealous if respondent talks with other men,” then the score is recoded as 1; otherwise 0. Similarly, for other values: (1) husband/partner accuses respondent of unfaithfulness; (2) husband/partner does not permit respondent to meet female friends; (3) husband/partner tries to limit respondent’s contact with family; (4) husband/partner insists on knowing where respondent is; (5) husband/partner doesn’t trust respondent with money; (6) ever been humiliated by husband/partner; (7) ever been threatened with harm by husband/partner; (8) ever been insulted or made to feel bad by husband/partner; (9) ever had bruises because of husband/partner’s actions; (10) ever had eye injuries, sprains, dislocations, or burns because of husband/partner; (11) respondent ever physically hurt husband/partner when he was not hurting her; (12) husband/partner: person who hurt respondent during a pregnancy; (13) respondent afraid of husband/partner most of the time, sometimes, or never.  A composite index was created using these dichotomous variables. The generated scores were categorized into low, moderate, and high using percentile value. The Cronbach α is .80. |
| Women’s educational attainment | The women’s educational attainment was recoded into 4 categories: 0=no education; 1=primary education; 2=secondary education; 3=higher and above |
| Educational attainment of head of the household | 0=no education; 1 =primary education; 2=secondary education; 3=higher and above |
| Number of antenatal care visit | 0 if women did not visit for antenatal care check-up, 1 if 1-4 visits; 2 if ≥4 visits |
| Place of delivery | 0 if women delivered in public facility; 1 if delivered in private facility; 2 if delivered at home |
| Height of women | If the height of women is <145.5 cm, then she is coded as very short; 145.6-149.9 as short; 150.0-155.0 as average; and ≥155.1 as tall |
| BMI of women | If the women BMI is below 18.5 then recoded as underweight; if BMI is between 18.5-24.9 =normal weight; if BMI is between 25.0-29.9 =Overweight; if BMI is 30 or greater =Obese. |
| History of anemia in woman | When the hemoglobin level was <11 g/dl, the women was diagnosed to have anemia. If the hemoglobin level was <8.0 g/dl, then she is categorized to have severe anemia; 8.0-10.9 g/dl as having moderate anemia; 11.0-11.9 g/dl as having mild anemia; ≥11.9 as nonanemic |
| Caste | The caste was categorized as follows: scheduled caste=1; scheduled tribe=2; other backward class=3; and none of them=4 |
| Religion | The religion was categorized as follows: Hindu=0; Muslim=1; Christian=2; and others=3 |
| Region | States were categorized into 6 regions: southern, central, north, eastern, northeastern, and western, which were coded as 0, 1, 2, 3, 4, and 5, respectively. |
| Wealth index | Poorest=1; poorer=2; middle=3; richer=4; richest=5; and high-wealth quintile=4 and 5 |
| Place of residence | Urban=0; rural =1 |
