# Supplementary material for: Alarming Trends of Cesarean Section—Time to Rethink: Evidence From a Large-Scale Cross-sectional Sample Survey in India
Source: J Med Internet Res. 2023 Feb 13;25:e41892. doi: 10.2196/41892 (PMC9972201; doi:10.2196/41892)
Supplement: Multimedia Appendix 2 [file jmir_v25i1e41892_app2.docx]

**Multimedia Appendix 2.** Socio-economic and demographic factors associated with type of delivery (elective versus emergency caesarean delivery) among women 5 years prior to survey, 2019-21, India.

| **Background Characteristics** | **Odd ratio for Elective C-section** | **Odd ratio for Elective C-section** |
| --- | --- | --- |
|  | **Unadjusted OR** | **Adjusted OR^1^** |
| **Age of women** | | |
| 15-19 | 1 | 1 |
| 20-24 | 1.08 [0.95,1.22] | 0.93 [0.59,1.47] |
| 25-29 | 1.23*** [1.09,1.39] | 0.98 [0.62,1.56] |
| 30-34 | 1.44*** [1.27,1.63] | 1.05 [0.65,1.69] |
| >35 | 1.58*** [1.39,1.81] | 1.26 [0.77,2.06] |
| **Educational attainment of women** | | |
| Illiterate | 1 | 1 |
| Primary | 1.02 [0.93,1.12] | 1.12 [0.85,1.48] |
| Secondary | 1.20*** [1.12,1.28] | 1.34* [1.07,1.68] |
| Sec and Higher | 1.23*** [1.14,1.32] | 1.31* [1.01,1.70] |
| **Educational attainment of head of household** | | |
| Illiterate | 1 | 1 |
| Primary | 0.97 [0.92,1.03] | 0.93 [0.78,1.12] |
| Secondary | 1.06** [1.02,1.11] | 0.98 [0.83,1.15] |
| Sec and Higher | 1.15*** [1.08,1.22] | 0.92 [0.74,1.15] |
| **Household Size** |  |  |
| 1 to 4 | 1 | 1 |
| 5 to 10 | 0.99 [0.95,1.03] | 0.96 [0.85,1.08] |
| > 11 | 0.95 [0.87,1.03] | 0.72 [0.49,1.04] |
| **Place of residence** |  |  |
| Urban | 1 | 1 |
| Rural | 0.86*** [0.83,0.90] | 0.96 [0.84,1.09] |
| **Religion** |  |  |
| Hindu | 1 | 1 |
| Muslim | 1.07** [1.02,1.12] | 0.96 [0.82,1.13] |
| Christian | 1.23*** [1.10,1.38] | 1.67** [1.14,2.43] |
| Others | 1.03 [0.93,1.14] | 1 [0.72,1.40] |
| **Caste** |  |  |
| SCs | 1 | 1 |
| STs | 1 [0.93,1.08] | 0.87 [0.68,1.12] |
| OBCs | 1.04 [0.99,1.08] | 0.92 [0.79,1.06] |
| None | 1.07** [1.02,1.13] | 0.94 [0.79,1.11] |
| **Region** |  |  |
| Southern | 1 | 1 |
| North | 0.74*** [0.70,0.78] | 0.68*** [0.58,0.81] |
| West | 0.92* [0.86,0.99] | 0.89 [0.70,1.15] |
| North-eastern | 0.74*** [0.70,0.78] | 0.85 [0.72,1.01] |
| Eastern | 1.07 [0.96,1.19] | 1.14 [0.82,1.58] |
| Central | 0.80*** [0.76,0.85] | 0.69*** [0.58,0.83] |
| **Wealth Index** |  |  |
| Poorest | 1 | 1 |
| Poorer | 1.22*** [1.13,1.32] | 0.98 [0.77,1.25] |
| Middle | 1.36*** [1.27,1.46] | 1.34* [1.06,1.71] |
| Richer | 1.48*** [1.38,1.59] | 1.43** [1.11,1.85] |
| Richest | 1.59*** [1.48,1.70] | 1.66*** [1.25,2.21] |
| **Partner human capital index** |  |  |
| Low | 1 | 1 |
| Moderate | 1.12 [0.99,1.27] | 0.97 [0.85,1.11] |
| High | 1.13 [0.99,1.30] | 0.98 [0.85,1.13] |
| **Parity** |  |  |
| 1 | 1 | 1 |
| 2 | 1.33*** [1.28,1.39] | 1.42*** [1.25,1.61] |
| 3+ | 1.32*** [1.25,1.40] | 1.38*** [1.15,1.66] |
| **Number of ANC visit** |  |  |
| No visit | 1 | 1 |
| 1 - 4 visits | 1.06 [0.95,1.18] | 1.23 [0.87,1.74] |
| >4 visits | 1.11* [1.00,1.24] | 1.09 [0.78,1.54] |
| **Height of women** | | |
| 150.0-155.0[Average] | 1 |  |
| <150.0[Short] | 0.88*** [0.85,0.92] |  |
| 155.1-239.9[Tall] | 1 [0.96,1.05] |  |
| **BMI of women** |  |  |
| Underweight | 1 | 1 |
| Normal Wt. | 0.90*** [0.85,0.95] | 0.84* [0.71,0.99] |
| Overweight | 1.04 [1.00,1.08] | 0.78*** [0.68,0.89] |
| Obesity | 1.22*** [1.15,1.30] | 0.97 [0.79,1.18] |
| **Anaemia level** | | |
| Severe | 1 | 1 |
| Moderate | 1.05 [0.91,1.22] | 0.78 [0.52,1.19] |
| Mild | 1.08 [0.94,1.25] | 0.68 [0.45,1.03] |
| Not anaemic | 1.15 [0.99,1.32] | 0.87 [0.57,1.31] |
| Refused/Not present |  |  |
| **Place of delivery** |  |  |
| Public | 1 | 1 |
| Private | 1 [0.96,1.03] | 0.97 [0.87,1.09] |
| Total | 44742 | 5232 |
| Note: ^1^Height of women and BMI were not controlled together due to collinearity; religion, caste, and region were controlled. Exponentiated coefficients; ^*^ *p* < 0.05, ^**^ *p* < 0.01, ^***^ *p* < 0.001; ^1^religion, caste, region and household size were controlled. The constitutional classification of caste was recoded, SCs: Scheduled Caste; STs: Scheduled Tribe, OBCs: Other Backward Caste, None: None of the SC, ST, and OBC | | |
